# Demands and stress before and during the COVID-19 pandemic of parents to children with autism spectrum disorder

**DOI:** 10.3389/fpsyg.2023.1212556

**Published:** 2023-10-03

**Authors:** Teresa Sartor, Sarah Sons, Olga Kunina-Habenicht, Heinrich Tröster, Jörg-Tobias Kuhn

**Affiliations:** Department of Rehabilitation Sciences, TU Dortmund University, Dortmund, Germany

**Keywords:** parental stress, parental demands, COVID-19 pandemic, autism spectrum disorder, ASD

## Abstract

**Introduction:**

Parents to children with Autism Spectrum Disorder (ASD) face diverse daily demands that can lead to stress. The aim of this study was to examine to which extent stress in parents to children with ASD can be explained by daily demands before and during the COVID-19 pandemic (after lockdowns; first half of 2022), and whether there are differences between the two time periods in this regard.

**Methods:**

Data from parents to children with ASD living in Germany from two independent questionnaire studies (before the pandemic: *N* = 168, during the pandemic: *N* = 105) were matched for comparability. Simple and multiple linear regression analyses were used to answer the research question.

**Results:**

Parental stress as well as all demands examined showed higher levels during the COVID-19 pandemic than before. Significant predictors of parental stress before and during the COVID-19 pandemic were (1) the daily demands to deal with the child’s problem behavior, (2) the restriction of one’s personal way of life, and (3) the challenge to cooperate with the partner. During the COVID-19 pandemic, the child’s problem behavior was particularly relevant. It was also found that the demand to deal with stigmatizing reactions did not explain parental stress during the COVID-19 pandemic whereas before the pandemic it had been a significant predictor.

**Discussion:**

Although parental stress and the demands of daily life increased during the pandemic, most of the stress can be explained by the same demands. It is suggested that the increased levels may be due to an increase in the child’s ASD symptomatology, which is why it is advisable to install therapeutic and care structures that prepare children with ASD for future crises.

## 1. Introduction

### 1.1. Parents to children with ASD: demands and stress

Numerous studies consistently show that parents to children with autism spectrum disorder (ASD) experience higher levels of stress than parents of neurotypical children (e.g., Hayes and Watson, 2013). During the COVID-19 pandemic, parents report high levels of stress compared to non-parents (e.g., Bujard et al., 2021). Among parents, parents to children with ASD are shown to experience even higher levels of stress (e.g., Kalb et al., 2021). This raises the question of which factors are responsible for the high level of stress examined and which daily demands placed on parents to children with ASD increased during the COVID-19 pandemic that may help explain their increased levels of perceived stress.

The demands that parents to children with autism spectrum disorder (ASD) face in their daily lives are enormous and multifaceted (O’Nions et al., 2018), even without a pandemic. These demands result in large part from the symptoms of ASD, which according to the DSM-5 are characterized by social communication impairments and restricted repetitive behavior and/or interests. Symptom severity is mapped on a continuum (American Psychiatric Association, 2013). The symptoms lead to impairments in the living arrangements of the child with ASD, which in turn requires a high level of support for the parents (Mancil et al., 2009). In what follows, we present everyday demands of parents to children with ASD that have been found to be significant in the literature (see Table 4, Tröster and Lange, 2019). Parents must deal with their child’s problematic behaviors (e.g., impaired communication) on a day-to-day basis, which presents challenges in daily interactions and parenting (*problem behavior of the child in education*; e.g., Ludlow et al., 2011), while maintaining a bond with their child, which can be particularly challenging for children with ASD (*parent–child-relationship*; e.g., Teague et al., 2019). Due to the symptoms typical of ASD, parents sometimes have to deal with stigmatizing reactions in public or in their social environment (*stigmatizing reactions in the social environment*; e.g., Woodgate et al., 2008; Vollmer et al., 2020). A commonly exhibited way of dealing with this demand is avoidance, which can lead to isolation (Minhas et al., 2015), and which in turn impacts social participation. On a day-to-day basis, it is difficult for parents to maintain social contacts on top of all family-related demands (e.g., Woodgate et al., 2008), as maintaining a social relationship involves effort and expectations (*social participation*; Diewald, 1991). Apart from maintaining social relationships outside the family, parents also need to maintain the relationship with their partner. They often face the challenge of perceiving themselves as lovers on top of the parental role (*cooperation with the partner*; e.g., Hock et al., 2012), because organizing family life can take a lot of time, especially for parents with children with ASD, as structures in everyday life are particularly important for them (*organization of family life*; e.g., Larson, 2006; Habermann and Kißler, 2022). This includes finding and coordinating appropriate professional support, such as making applications, organizing and keeping appointments (*professional support*; e.g., Vollmer et al., 2020). In addition, parents are left with little time and space for their personal needs, such as hobbies or their own careers (*personal way of life*; e.g., Hoogsteen and Woodgate, 2013).

These numerous high demands are reflected in the stress experience of parents to children with ASD. According to Lazarus’ transactional stress theory, stress must be viewed in terms of a reciprocal person-environment relationship, focusing on cognitive appraisals and coping in the stress process (Lazarus and Launier, 1978; Lazarus and Folkman, 1984; Lazarus, 1999). Using Abidin’s (1995) parenting stress model, which “represents an explication of a specific application of Lazarus and Folkman’s general theory” (Abidin, 1992, p. 410), parental stress can be accounted for as follows: Parental stress occurs when parents are confronted with demands in their parenting that they cannot cope with using their available resources (Abidin, 1992; Tröster, 2011). In the parenting stress model, two main sources of stress are distinguished: first, the parent domain is outlined, which includes the stress that arises from the limitation of parental functions, making it more difficult for parents to cope with the demands of their parenting role, such as a dysfunctional partnership, social isolation, or unstable health; second, the child domain is described as the stress that results from a child’s characteristics and behaviors, such as a limitation in adaptability or the child’s hyperactivity or mood (Abidin, 1995). Especially in the child domain, parents to children with ASD show higher levels of stress than parents to children without disabilities (Hoffman et al., 2009; Tröster and Lange, 2019) and likewise than parents to children with other disabilities (e.g., intelligence impairment or Down syndrome, Hayes and Watson, 2013).

### 1.2. Parents to children with ASD during the COVID-19 pandemic

Although parents were generally more stressed during the pandemic (e.g., Bujard et al., 2021), studies show that parents to children with ASD, in contrast to parents with neurotypical children, experience significant stress during the pandemic (Corbett et al., 2021; Kalb et al., 2021) and these parents report more symptoms of depression and anxiety than parents to typically developing children (Wang et al., 2021). This may be related to the specific demands that parents experience as a result of their child’s autism diagnosis, which may be compounded by the general demands of the COVID-19 pandemic.

Since the beginning of the COVID-19 pandemic in Germany in March 2020 (World Health Organization, 2020), most people’s daily lives have been affected by health-related pandemic measures, e.g., restriction of social contacts (Federal Ministry of Health, 2021). For parents, the closure of childcare facilities and educational institutions was particularly challenging, requiring children to be cared for and schooled at home (OECD, 2021; Stadheim et al., 2022). The results of a study from 2020 showed that parental satisfaction during the pandemic in Germany was lower than before the pandemic in all areas surveyed, such as childcare or family life (Huebener et al., 2020), and parents felt more stressed than non-parents (Bujard et al., 2021). Broadening the view, similar findings emerged in other countries. In the American Psychological Association 2020 study, parents were found to be significantly more stressed than non-parents during the pandemic, especially in the pandemic-related aspects. The group of children with ASD and their families appeared to be particularly impacted by the pandemic because of its potential to exacerbate ASD symptoms (Bellomo et al., 2020; White et al., 2021). In a study in Germany and Austria by Isensee et al. (2022), almost 50% of parents reported an increase in symptoms in their child with ASD already at the onset of the pandemic. The study by Vasa et al. (2021) also reported that over half of the children with ASD either developed new psychiatric symptoms during the pandemic or had their pre-existing psychiatric conditions exacerbated. Colizzi et al. (2020) showed that more than one-third of children with ASD had their preexisting behavioral problems aggravated during the pandemic. An increase in mood symptoms, maladaptive behaviors (Stadheim et al., 2022) and sleep disturbances, a reinforcement of ritualized behaviors, and a decrease in motivation for social interaction were also evident in children with ASD (Latzer et al., 2021). In addition, children with ASD showed increased pandemic anxiety at the beginning of the pandemic (Hall et al., 2023). According to White et al. (2021), the greater expression of ASD symptoms could be explained by the frequent or prolonged interruption of therapy. Amplification may also result directly from the day-to-day realities of the pandemic. Children with ASD had particular difficulties in adapting to the new daily routines and patterns that the pandemic brought with ever-changing regulations (Latzer et al., 2021); at the same time, their parents longed for stability and normalcy (Stadheim et al., 2022). The increased severity of ASD in turn has an impact on parental stress: the more severe the symptoms of ASD, the more stressed parents feel during the pandemic (Manning et al., 2021) and the more intrafamilial conflicts increase (e.g., physical violence or aggression; Isensee et al., 2022). Results show that parents to children with ASD report difficulty in coping with the daily demands regarding the child in particular (Colizzi et al., 2020).

It can be summarized that parents to children with ASD were highly stressed and the numerous demands in their daily lives that they had to cope with were intensified by the COVID-19 pandemic. Most studies to this point relate to the lockdown phases of the COVID-19 pandemic and not (yet) to the immediate post-strict phase period (see Procedures section for details on chronology). The aim of this study is to fill this research gap to some extent and to help clarify the increased stress experienced by parents to children with ASD during the COVID-19 pandemic, post-lockdown period, by answering the question of which demands in daily life explain this increased stress. Based on existing results surveyed during the pandemic (e.g., Colizzi et al., 2020), we hypothesize research that daily demands and parental stress during the pandemic are higher than before (H1). Due to the increase in symptoms during the pandemic (e.g., Manning et al., 2021; Isensee et al., 2022), we postulate as our second research hypothesis (H2) that parental stress is mainly explained by the need to deal with the child’s behavior on a daily basis. Similarly, living a personal lifestyle (H3) and the need to organize family life (H4) are hypothesized to have a predictive influence due to the increased care needs of parents as a result of closed care institutions (e.g., Huebener et al., 2020; Stadheim et al., 2022). Two samples were compared to answer the research question. Details of the statistical analyses will be outlined below.

## 2. Method

### 2.1. Procedures

Two samples were used in this study: one sample of parents to children with ASD before and one during the COVID-19 pandemic in Germany. The first sample (hereafter referred to as S1) was collected as part of the research project “ELKASS” (Parents to children with autism spectrum disorders: Demands, stress and resources; Tröster and Lange, 2019) from December 2015 to March 2016. The second sample (hereafter S2) was collected between April and June 2022 during the ongoing COVID-19 pandemic. At the end of March 2022, according to the government, the onset of a new phase of the pandemic began: the removal of most protective measures (Federal Government of Germany, 2022a). In most regions, there were no general contact restrictions (e.g., regarding the number of people allowed to meet in private) during the survey period, educational institutions were open, and most recreational facilities were open (Federal Government of Germany, 2022b). Differences in the survey method arose in that S1 was surveyed exclusively with a paper-pencil survey at the ten cooperating autism therapy centers that were requested via the German Autism Association. In S2, in addition to printed questionnaires, an online questionnaire was shared among distributors of therapy centers, institutions, and social media groups to be independent of renewed closures of institutions or stricter contact restrictions. All paper questionnaires were sent to the researcher in anonymized covers via the cooperating institutions Some autism therapy centers from S1 also sent the questionnaire to their members at the S2 time point. Therefore, it cannot be excluded that individual participants took part in both surveys. Due to the assurance of anonymity, participating parents were not asked about the institution they visited. An official review by an ethics committee was carried out in advance, the result of which being a positive vote without any restrictions.

### 2.2. Participants

S1 is composed of *N* = 168 parents to children with ASD who had just started support at an autism therapy center. In S2, *N* = 105 parents to children with ASD participated during the COVID-19 pandemic. The subjects were largely mothers (S1 = 86.9%; S2 = 96.2%) who reported to be of German nationality (S1 = 85.1%; S2 = 93.3%). The age of the parents interviewed ranged from 24 to 62 years (S1: *M* = 39.43, *SD* = 6.58; S2: *M* = 39.95, *SD* = 5.5) and they had between one and six children (S1: *M* = 1.92, *SD* = 0.88; S2: *M* = 2.12, *SD* = 0.91). Their child with ASD was between 2 and 12 years old (S1: *M* = 8.0, *SD* = 2.67; S2: *M* = 7.91, *SD* = 2.61) and most of them were male (S1: 87.5%; S2: 74.3%). Additional sociodemographic information of the parents as well as the diagnoses of the child with ASD can be found in Table 1. Retrieved information on the situation at home during the pandemic is shown in Table 2.

**Table 1:** Socio-demographic data.

	S1		S2	
	*n*	*%*	*n*	*%*
**Diagnosis of the child with ASD**				
Childhood autism (ICD-10, F84.0)	61	36.3	37	35.2
Atypical autism (ICD-10, F84.1)	20	11.9	21	20.0
Asperger syndrome (ICD-10, F84.5)	80	47.6	35	33.3
Pervasive developmental disorder, unspecified (ICD-10, F84.9)	3	1.8	4	3.8
Autism spectrum disorder (DSM-5, 299.0)	4	2.4	8	7.6
**Parenting situation**				
Single parent	40	23.8	13	12.4
Two-parent families	128	76.2	92	87.6
**Employment situation**				
Working full-time	33	19.6	15	14.3
Working part-time	70	41.7	54	51.4
Studying	5	3.0	3	2.9
At home (e.g., parental leave, housewife/househusband)	60	35.7	33	31.4
**Educational attainment**				
University degree	46	27.4	41	39.0
High school	48	28.6	28	26.7
Intermediate secondary school	52	31.0	31	29.5
General secondary school	16	9.5	5	4.8
No qualification	6	3.6	0	0.0
**Professional support measures** ^**1**^				
None	78	46.4	48	45.7
Educational counseling center	28	16.7	19	18.1
Parent training	24	14.3	17	16.2
Self-help group	21	12.5	19	18.1
Other (e.g., family assistance, psychotherapy, social pediatric center)	42	25.0	26	24.8

Sociodemographic data for the two samples S1 = 168 (before the pandemic) and S2 = 105 (during the pandemic). Data differ due to missing values.^1^Multiple selections were possible.

**Table 2:** Situation at home during the pandemic.

		S2	
		*n*	*%*
**Places of work during the COVID-19 pandemic** ^**1**^			
Mainly/exclusively at the workplace		42	40.0
Balanced		10	9.5
Mainly/exclusively from home		14	13.3
**Time child had to be cared for at home during pandemic**			
Up to 1 month		9	8.6
Up to 4 months		38	36.2
Up to half a year		25	23.8
Over half a year		28	26.7
**Childcare at home during pandemic** ^**2**^			
None		6	5.7
Other parent/partner		30	28.6
Family/friends/acquaintances		21	20.0
Interviewed parent him−/herself (e.g., working from home, parental leave)		104	99.1
Urgent care in facilities (e.g., schools and daycare centers)		21	20.0
Other (e.g., self-paid care, school assistance)		5	4.8

Retrieved information about the situation at home during the pandemic from S2 (*n* = 105). Data differ due to missing values.^1^Only employed persons;^2^Multiple selections were possible.

### 2.3. Materials

S1 and S2 received the same questionnaire. The questionnaire measured parenting stress and parenting demands in everyday life. In addition, the questionnaire collected sociodemographic information. In S2, additional pandemic-related information was collected.

#### 2.3.1. Parental stress

The stress of parents to children with ASD was assessed with the “Eltern-Belastungs-Inventar” (EBI; Tröster, 2011), which is a German version of the Parenting Stress Index (PSI) of Abidin (1983). The 48 items are assigned to a total of 12 subscales (see Table 3), which in turn belong to either the child domain (five subscales) or the parent domain (seven subscales). The child domain is defined by the stress resulting from the child’s behavior and characteristics. The parent domain is defined by the stress resulting from the limitations of parental functions needed to cope with the educational demands. The items were answered by the parents on a five-point scale (1 = “does not apply at all” to 5 = “applies exactly”). Cronbach’s alpha was very good for the normative sample in the two domains (child domain: *α* = 0.91, parent domain: *α* = 0.93; Tröster, 2011, p. 32). The internal consistencies as well as an example item for each subscale are shown in Table 3.

**Table 3:** Parent and child domain subscales of the EBI.

Parent domain	Example item	α
Isolation	*Since I became a mother/father, it is more difficult for me to make new contacts.*	*0.63*
Depression	*It depresses me when I realize that I am reacting irritably to my child.*	*0.75*
Health	*Lately I do not feel as fit and efficient as I used to.*	*0.75*
Spouse	*Because of the child, some problems arose in my partnership.*	*0.80*
Role restriction	*In order to do justice to my child, I have to limit myself more than I imagined.*	*0.82*
Parental attachment	*In some situations, I wish I could better empathize with what is going on in my child.*	*0.61*
Parental competence	*Some things in raising my child are harder for me than I expected.*	*0.83*
**Child domain**		
Adaptability	*My child sometimes has a hard time adjusting to changes in routine or home environment.*	*0.77*
Acceptability	*My child does some things that bother me.*	*0.70*
Demandingness	*My child is doing some things that are taking a toll on me.*	*0.68*
Mood	*When playing, my child is often impatient and gets angry quickly.*	*0.70*
Distractibility/Hyperactivity	*My child has more difficulty than other children in concentrating and paying attention.*	*0.75*

Presented are the subscales of the EBI, each with an example item and the internal consistency (Cronbach’s alpha) for each subscale from the normative sample (Tröster, 2011). Each subscale includes four items.

#### 2.3.2. Parental demands in everyday life

The parental demands in everyday life were surveyed with the scale by Tröster and Lange (2019). The scale measures behavioral, cognitive, and social–emotional demands of parents, which are divided into eight subscales (see Table 4). A total of 41 items are answered on a four-point scale from 1 = “never / rarely” to 4 = “very often” according to how often a situation occurs in everyday life. Because the instrument is not yet standardized, the internal consistencies of the present samples are reported. The overall scale had very good reliability in both samples (S1: *α* = 0.92, S2: *α* = 0.94). Example items are illustrated in Table 4, as well as the good internal consistency of the individual subscales. The two subscales parent–child relationship and professional support were excluded from all analyses due to their unacceptable Cronbach’s alpha.

**Table 4:** Subscales of the “Parental demands in everyday life” scale.

Demands	Example item	*α* S1	*α* S2
Problem behavior of the child in education (BC), 12 items	*My child has a hard time adjusting to new situations.*	*0.72*	*0.79*
Stigmatizing reactions in the social environment (SR), 7 items	*I have to justify my child’s conspicuous behavior to other people.*	*0.89*	*0.88*
Social participation (SP), 2 items	*I have to limit my social contacts.*	*0.82*	*0.86*
Cooperation with the partner (CP), 4 items	*There is little time for joint activities with my partner.*	*0.78*	*0.85*
Organization of family life (FL), 5 items	*Joint activities with the family are difficult to realize.*	*0.74*	*0.85*
Personal way of life (PL), 5 items	*I do not have the time to deal with other things.*	*0.87*	*0.90*
Parent–child relationship (PCR), 4 items^1^	*I find it difficult to establish emotional closeness with my child.*	*0.43*	*0.48*
Professional support (PS), 2 items^1^	*Coordinating with teachers, therapists,* etc. *on the proper care for our child is difficult.*	*0.37*	*0.24*

Presented are the subscales of the “Parental demands in everyday life” scale (Tröster and Lange, 2019), each with an example item and the internal consistency (Cronbach’s alpha) for each subscale in the two samples of the present study. The two subscales parent-child relationship and professional support were excluded from all analyses due to their unacceptable Cronbach’s alpha.

### 2.4. Data analyses

R version 4.2.1 (R Core Team, 2021) was used for all analyses.

#### 2.4.1. Matching

In a first step, S1 and S2 were matched for better unbiased comparison, using the following sociodemographic data of parents and children with ASD: age of parent interviewed, gender of parent, parenting status (single parent or not), educational achievement, employment situation, number of children, age of child with ASD, gender of child with ASD, diagnosis of child with ASD, comorbidity of child with ASD. A propensity score matching (PSM; Pan and Bai, 2015) with full matching was applied and the package “MatchIt” (Ho et al., 2011) was used.

#### 2.4.2. Multiple imputation

For the dependent variables (parental stress) and the independent variables (demands) together, a total of 5% of the values were missing. These were treated under the assumption of missing at random (MAR) and imputed using the R-package “mice” with a number of 20 imputations and 10 iterations per imputation (van Buuren and Groothuis-Oudshoorn, 2011). For the imputation, the same socio-demographic data were used as for the matching (see above) and additionally the variables included in the analysis (stress and demands).

#### 2.4.3. Regression analyses

To determine whether differences exist in the demands or stress of parents to children with ASD before and during the COVID-19 pandemic, the two matched groups (S1 and S2, independent variables) were compared in the demands and the two stress domains (dependent variables) using simple linear regression analyses. Multiple linear regression analyses were then used to examine which demands are significant in elucidating parental stress (main effects), and whether differences in predictor importance between the two groups were apparent (interaction effects). In this analysis, the stress domains were used as dependent variables and the different demands as independent variables.

## 3. Results

Before the regression analyses could be used to answer the research question, the two samples were matched for better comparability. Propensity score matching using the 1:1 nearest neighbor method did not yield acceptable results, such that the full matching method (Hansen, 2004; Stuart and Green, 2008) with distance gml and probit regression was applied. This method could achieve a satisfactory balance: After matching, all standardized mean differences for the covariates were below 0.18. According to Cochran and Rubin (1973), the difference should be lower than 0.25, which is thus fulfilled. Further, after propensity score matching, no significant differences in sociodemographic data used for matching (see chapter Statistical Analysis) between S1 and S2 remained (*p* > 0.05). Full matching allowed all cases from the sample to be included, and no cases had to be discarded.

The results of the simple linear regression analyses with demands and stress as the dependent variable and sample as the binary independent variable showed significant differences in all demands and both stress domains concerning to the time of the survey (see Table 5). The sample surveyed during the COVID-19 pandemic reported both higher demands in daily life and higher parental stress. According to Cohen’s classification (Cohen, 1988), the effect sizes are in the medium range, with the exception of the demand social participation, where a small effect was found, and the demand problem behavior of the child, where a large effect was present. Tables 6, 7 present the results of multiple regressions with stress as the dependent variable and demands as the independent variables. In the parent domain, significant main effects were shown in the demands of stigmatizing reactions, personal way of life, cooperation with the partner, and problem behavior of the child (adj. *R*^2^ = 0.59). In the demand stigmatizing reactions, an additional significant interaction effect was found, suggesting that the significance of this predictor differed between the two samples. Further testing showed that the demand stigmatizing reactions was only a significant predictor of stress in the parent domain during the COVID-19 pandemic, but no longer during the pandemic (*B* = 0.50, *p* > 0.05). In the child domain, similar significant main effects were found in the following demands: stigmatizing reactions, cooperation with the partner, and problem behavior of the child (adj. *R*^2^ = 0.50). The negative interaction effect in the demand stigmatizing reactions was also found, similar to the parent domain. The main effect during the COVID-19 pandemic was not significant either upon further analysis (*B* = 0.15, *p* > 0.05). In addition, a second significant interaction effect was found in the demand problem behavior of the child. It can be inferred from the positive sign that this predictor is especially important during the pandemic for the explanation of the stress.

**Table 5:** Results of simple linear regression analyses.

	*B*	*SE*	*t*	*p*	*d*	*R^2^ adj*
Stress parent domain	0.39	0.09	4.17	<0.001	0.55	0.06
Stress child domain	0.37	0.08	4.60	<0.001	0.60	0.07
Organization of family life (FL)	0.55	0.09	6.24	<0.001	0.55	0.13
Stigmatizing reactions (SR)	0.26	0.09	3.00	0.003	0.55	0.03
Social participation (SP)	0.24	0.11	2.09	0.037	0.44	0.01
Personal way of life (PL)	0.26	0.10	2.62	0.009	0.51	0.02
Cooperation with the partner (CP)	0.36	0.10	3.72	<0.001	0.50	0.05
Problem behavior of the child (BC)	0.38	0.06	6.34	<0.001	0.81	0.13

Dependent variables: stress and demands; independent variable: sample (S1 and S2).

**Table 6:** Results of multiple regression analysis: parent domain.

	*B*	*SE*	*t*	*p*
Sample	0.18	0.37	0.48	0.634
Organization of family life (FL)	−0.04	0.08	−0.51	0.610
Stigmatizing reactions (SR)	0.33	0.08	4.03	<0.001
Social participation (SP)	−0.11	0.08	−1.37	0.172
Personal way of life (PL)	0.54	0.09	5.66	<0.001
Cooperation with the partner (CP)	0.34	0.07	5.32	<0.001
Problem behavior of the child (BC)	−0.24	0.10	−2.36	0.018
Sample × FL	0.08	0.13	0.67	0.503
Sample × SR	−0.26	0.12	−2.08	0.037
Sample × SP	0.25	0.13	1.86	0.063
Sample × PL	−0.28	0.15	−1.84	0.066
Sample × CP	−0.05	0.10	−0.50	0.619
Sample × BC	0.21	0.18	1.19	0.233

Dependent variables: stress in the parent domain; independent variables: demands and sample (S1 and S2); with interaction effects.

**Table 7:** Results of multiple regression analysis: child domain.

	*B*	*SE*	*t*	*p*
Sample	−0.19	0.36	−0.54	0.587
Organization of family life (FL)	0.05	0.09	0.61	0.545
Stigmatizing reactions (SR)	0.35	0.08	4.28	<0.001
Social participation (SP)	0.06	0.09	0.73	0.464
Personal way of life (PL)	0.01	0.09	0.14	0.889
Cooperation with the partner (CP)	0.20	0.07	2.96	0.003
Problem behavior of the child (BC)	0.24	0.10	2.41	0.016
Sample × FL	−0.01	0.13	−0.06	0.954
Sample × SR	−0.33	0.12	−2.71	0.007
Sample × SP	−0.18	0.13	−1.40	0.161
Sample × PL	0.25	0.15	1.73	0.084
Sample × CP	−0.08	0.10	−0.78	0.438
Sample × BC	0.35	0.17	2.05	0.040

Dependent variables: stress in the child domain; independent variables: demands and sample (S1 and S2); with interaction effects.

## 4. Discussion

The present study addressed the questions of the extent to which stress in parents to children with ASD before and during the COVID-19 pandemic is associated with their daily demands, and whether there are differences between the two time points. For this purpose, two non-longitudinal matched samples were compared before and during the pandemic.

The results show higher levels of all demands examined and both stress domains at time S2 than at S1. This confirms our H1. Thus, parents to children with ASD experience higher everyday demands and experience more stress during the COVID-19 pandemic. This finding is consistent with other study results (e.g., Colizzi et al., 2020). However, the fact that all demands are higher does not mean they cause the increased stress levels during the COVID-19 pandemic. The results show that there are few significant differences in predictors of stress before and during the pandemic. Differences appeared (1) in both stress domains in the demand stigmatizing reactions, which was no longer important for stress during the pandemic, and (2) in the demand problem behavior of the child, which was especially important for stress in the child domain during the pandemic, which confirms H2. Findings from other studies showed that ASD symptomatology increased in children with ASD during the pandemic (e.g., Latzer et al., 2021; Vasa et al., 2021; Isensee et al., 2022; Stadheim et al., 2022). This may serve as an explanation for the present finding on the problem behavior of the child, because it can be assumed that the demand of having to deal with the child’s autism-specific behavior in daily life increases when symptoms increase, resulting in more stress and thus supporting the findings of Manning et al. (2021). The pandemic may have increased problematic behaviors, e.g., due to a lack of therapy sessions (White et al., 2021). Further, constant changes of the pandemic (e.g., parents working from home; Latzer et al., 2021) may have increased problematic behaviors. Rehearsed responses to the child’s problem behavior as well as routines in daily life can be difficult to maintain due to the constantly changing infection control regulations (e.g., closure of institutions or contact restrictions), which is why therapeutic support seems particularly necessary at a time like that. Stadheim et al. (2022) identify deficits in educational and therapeutic service systems for children with ASD and suggest a hybrid form for service delivery to ensure continuity. A practice of digital appointments can prepare the child for further crises and also ensure regular therapy if the child cannot appear on site for other reasons (e.g., transportation problems, vacation). This requires structures such as technical equipment on both sides, service and families at home (Isensee et al., 2022), as well as trained media competence. Such structures need to be discussed politically among service providers.

In addition to the child’s problem behavior as a demand, only one other difference emerges between the demands that elucidate parental stress: the demand in daily life to cope with stigmatizing reactions emerges as an important predictor of stress in both the child and parent domains before the pandemic. In contrast, this demand no longer affects stress during the COVID-19 pandemic, although the demand - like all others - shows higher levels than before the pandemic. In response to stigmatizing their own child with ASD, parents often use avoidance by isolating themselves (Minhas et al., 2015). As a demand, stigmatizing reactions in the social environment would be expected to no longer be relevant to that extent due to reduction in contact with fellow individuals during the pandemic. The result of an increased level of stigmatizing reactions can be explained by increased symptoms, in that the child’s behavior is more noticeable to strangers and the parents therefore fear more stigmatizing reactions, because higher severity of disability is more associated with rejection (Miller et al., 2009). Despite the increased demand to manage these reactions, this does not lead to more stress during the pandemic. It is possible that parents focused on other demands and paid less attention to other people’s reactions. In relation to the stress models of Lazarus and Folkman (1984) and Abidin (1992), it is conceivable that parents may have developed new strategies to deal with this stigma during the pandemic (e.g., impression management; Voysey, 1972). Accordingly, they would consider their resources or strategies to be sufficient to handle this demand. The assumption that parents developed new strategies to deal with stigmatizing reactions from others during the pandemic would need to be investigated in further studies. In addition, it should be investigated what these strategies are and whether they continue to protect parents in the long term, after the pandemic.

Apart from that, no differences were found in which demands clarify the stress. The demands during the pandemic are higher, but before and during the pandemic the same demands are responsible for the emergence of parental stress. In addition to the two demands mentioned above (problem behavior of the child and stigmatizing reactions), the limitation in personal way of life in both the child and parent domains is explanatory for stress at both time points. The result confirms H3. Due to the high level of caregiving required, time for personal activities and taking on a role other than parent of a child with ASD can be very difficult (Hoogsteen and Woodgate, 2013). During the pandemic, there are many restrictions on recreational activities and, thus, families spend most of their time at home. It is conceivable that especially parents to children with ASD want to minimize the risk of infection as much as possible since their child belongs to the vulnerable group of the COVID-19 pandemic (Karpur et al., 2021). A hospital stay for the child in a foreign environment and a lack of routines is unthinkable, which brings about even stricter isolation. System-changing measures, such as emergency care systems (possibly digital) during crises should be politically reconsidered. These would need to be practiced over time and established as a fixed structure so that children with ASD can benefit from this offer, rather than experiencing a sudden change that they have difficulties coping with.

Stress in the parent domain is additionally influenced by the demand of cooperating with a partner both before and during the COVID-19 pandemic. Parents to children with ASD must coordinate care and nurturing of the child on a large scale (Hock et al., 2012). The challenge of fulfilling the role as (parenting) partner and of coordinating and agreeing with the partner about the child’s upbringing and care seems to be a present demand in the daily life of parents to children with ASD, leading to stress in the parent domain. Isolation and quarantine periods may have led to increased potential for conflict in the partnership (Waddel et al., 2021; Overall et al., 2022). In the balancing act of caring for children and working from home, there is less time to discuss issues calmly as a couple. During the pandemic, many parents cared for children during the day and pursued their professional activities from home in the evenings (Beno, 2021). In the therapeutic setting, parents should be enabled to perceive the cooperation with the partner as a resource and not as a burden. If parents perceive themselves as a parenting alliance (Cohen and Weissmann, 1984) and master the challenges of daily life with a child with behavioral problems together, this can strengthen the relationship (Gabriel and Bodenmann, 2006). In turn, a good partnership relationship can reduce stress of parents to children with ASD (e.g., Siman-Tov and Kaniel, 2011).

All other demands surveyed showed higher levels during the pandemic than in the before-pandemic sample, but they had no relevance for stress. Establishing and maintaining social contacts seems to have become more difficult in times of the pandemic, presumably due to regulations and recommendations to restrict contact and by the self-imposed isolation due to the child’s risk status. It has not been demonstrated that stress results from this demand. Apart from all the positive aspects, establishing and maintaining social relationships costs time and energy (Diewald, 1991). During the pandemic, the focus may have shifted to more pressing issues.

H4 could not be confirmed by the present results. Although family organizational demands were reported to be higher during the pandemic, they do not explain the increased stress levels of parents. In particular, due to the many ever-changing pandemic regulations, it could be difficult to organize family life, such as spending time together as a family. It may be that the immediate reward – family time together (Stadheim et al., 2022) – is worth organizing and therefore does not directly cause stress. For children with ASD, routine is an important building block in daily life (Bagatell, 2015), so if it is well organized, the child also benefits and presumably their behavior reflects that.

In general, the results show that some increased demands during the pandemic led to stress and thus, according to the stress models mentioned above (Lazarus and Folkman, 1984; Abidin, 1992), resources appeared to be insufficient to cope with the demands. It needs to be investigated in subsequent research which resources exactly are overused in this context. Possible coping resources that have already been shown to be effective in the literature among parents to children with ASD, such as parental self-efficacy (e.g., Giallo et al., 2013; Weiss et al., 2013; Tröster and Lange, 2019), social support (e.g., Siman-Tov and Kaniel, 2011; Robinson and Weiss, 2020; Weiss et al., 2021), or functional coping strategies, such as active coping (e.g., Wang et al., 2013), should be taken into account in further research.

## 5. Limitations

Although propensity score matching was a step in the right direction toward comparability, there is no real longitudinal study available and the results should not be interpreted causally as such. For S2, no information is available on the stress level before the pandemic. Therefore, it is possible that the parenting stress or demands were already higher in S2 than in S1 before the pandemic. Nevertheless, the exploratory results ran important findings.

This study focused only on the general demands of the daily lives of parents to children with ASD as possible predictors for elucidating parental stress. Specific predictors directly resulting from the COVID-19 pandemic could have been included in the analyses. For example, the additional teaching role that parents had to take on during school closures. According to previous studies, this appears to be a major challenge for parents and a potential stressor (e.g., Eckert and Kamm Jehli, 2021). In addition, the inclusion of heterogeneity and severity of ASD symptoms would be useful in future studies to provide a more nuanced view, as severity has an impact on parental stress, as shown, for example, in the study by Manning et al. (2021).

An important next step would be to use the influence of parental coping strategies and resources for clarification to allow a more comprehensive picture of reality. In general, stress research is always tainted with a negative view of the families studied. Therefore, resources of parents as well as resources of children with ASD should be focused on. Thus, not only challenges but also opportunities of the COVID-19 pandemic would be discussed. It could be assumed that children with ASD may also benefit from the changing conditions of the pandemic, such as being able to attend classes from home as a safe place (Nashef, 2020) or no longer having to sit on a stimulus-crowded school bus. Fumagalli et al. (2021) note that children with ASD report a more positive mood during the first few weeks of the pandemic compared to neurotypical children. Many children with ASD and their families may also benefit from slowing down their daily routines (Eckert and Kamm Jehli, 2021). In the study by Stadheim et al. (2022), some parents reported and valued a strengthened cohesion within their family during the pandemic. In the period after the pandemic, new structures in the home environment could be pursued. Empirical studies need to pick up here and investigate which factors are helpful for families to maintain this sense of cohesion. During the intensive time spent together throughout the pandemic, new structures were formed that many families would not want to miss (Pavlopoulou et al., 2020). In the post-pandemic period, the new structures in the home environment could be followed up. Empirical studies need to pick up here and investigate which factors are helpful for families to maintain these positive aspects.

## 6. Practical implications

In summary, the COVID-19 pandemic can be described as a time of crisis for parents to children with ASD. Both daily demands and parental stress were heightened. Studies show that families with members with ASD did not feel adequately addressed during the pandemic (Pavlopoulou et al., 2020). Support to reduce stress-inducing demands should first prioritize working with families. Child care support should be secured so that stress caused by personal limitations as well as the lack of opportunity to work on the partner relationship can be reduced. To mitigate reinforcement of the child’s problem behavior, regular therapeutic support should also be available during a pandemic. Alternatives (such as online therapy sessions) have already been developed during the COVID-19 pandemic (Kaku et al., 2021). In addition to focusing on the increased demands themselves due to the pandemic, ways how these demands are handled should also be addressed since, among other reasons, it is sometimes difficult or impossible to address the demands directly (e.g., the pandemic containment regulations that cannot be influenced). Therefore, families should receive therapeutic guidance on how to cope with and manage the new or increased demands or consequences of the pandemic (e.g., how to repeatedly practice new routines with the child), so that they are prepared for future times of crisis (Stadheim et al., 2022). The question of which coping strategies work under which circumstances for parents to children with ASD for the pandemic-related demands so that they experience less stress should be empirically evaluated.

## Data availability statement

The datasets presented in this article are not readily available because the data will be used for further analyses and publications. Requests to access the datasets should be directed to teresa.sartor@tu-dortmund.de.

## Ethics statement

The studies involving humans were approved by “Gemeinsame Ethikkommission der Fakultäten 11–17 der TU Dortmund.” The studies were conducted in accordance with the local legislation and institutional requirements. The participants provided their written informed consent to participate in this study.

## Author contributions

TS: idea for writing this manuscript, data collection S2, data curation S1 and S2, data analysis, writing and revision of the draft (lead). SS: data collection S1, data curation S1, and revision of the draft. OK-H: project administration S2 and revision of the draft. HT: project administration S1, data collection S1, data curation S1, and revision of the draft. J-TK: supervision, project administration S2, and revision of the draft. All authors contributed to the article and approved the submitted version.
